# Combining data augmentation and deep learning for improved epilepsy detection

**DOI:** 10.3389/fneur.2024.1378076

**Published:** 2024-04-03

**Authors:** Yandong Ru, Zheng Wei, Gaoyang An, Hongming Chen

**Affiliations:** ^1^School of Information Engineering, Zhejiang Ocean University, Zhoushan, China; ^2^Key Laboratory of Oceanographic Big Data Mining & Application of Zhejiang Province, Zhejiang Ocean University, Zhoushan, China; ^3^School of Electronics and Information Engineering, Heilongjiang University of Science and Technology, Harbin, China

**Keywords:** epilepsy detection, data augmentation, attention mechanism, one-dimensional convolutional neural network, gated recurrent unit

## Abstract

**Introduction:**

In recent years, the use of EEG signals for seizure detection has gained widespread academic attention. Aiming at the problem of overfitting deep learning models due to the small number of EEG signal data during epilepsy detection, this paper proposes an epilepsy detection method that combines data augmentation and deep learning.

**Methods:**

First, the Adversarial and Mixup Data Augmentation (AMDA) method is used to realize the data augmentation, which effectively enriches the number of training samples. To further improve the classification accuracy and robustness of epilepsy detection, this paper proposes a one-dimensional convolutional neural network and gated recurrent unit (AM-1D CNN-GRU) network model based on attention mechanism for epilepsy detection.

**Results and discussion:**

The experimental results show that the performance of epilepsy detection achieved by using augmented data is significantly improved, and the accuracy, sensitivity, and area under the subject’s working characteristic curve are up to 96.06, 95.48%, and 0.9637, respectively. Compared with the non-augmented data, all indicators are increased by more than 6.2%. Meanwhile, the detection performance was significantly improved compared with other epilepsy detection methods. The results of this research can provide a reference for the clinical application of epilepsy detection.

## 1 Introduction

Epilepsy is the second largest neurological disorder in the world, a chronic disease caused by sudden abnormal discharge of brain neurons leading to transient brain dysfunction (1). There are approximately 50 million patients worldwide, and patients may experience injuries or even life-threatening emergencies during seizures, causing great psychological stress and work-life difficulties (2). In clinical practice, electroencephalograph (EEG) is one of the most common and important examination tools used in the diagnosis and treatment of epilepsy (3). As a non-invasive EEG activity detection tool, it records a large amount of EEG information, which is beneficial for doctors to identify the lesion and carry out effective treatment. However, due to the uncertainty of epileptic seizures, which cannot be observed in a short period, EEG signals need to be observed and captured over a long period, which is an inefficient and time-consuming process. Therefore, the automatic detection of epileptic EEG helps reduce the workload of medical workers, and it becomes particularly important to further promote the research on the automatic detection of epileptic EEG (4).

The use of EEG for epileptic detection has received extensive academic attention over the past decades, and the research methods mainly include machine learning and deep learning. Among them, machine learning requires artificial design algorithms to obtain EEG signal features, and then combine with related classifiers to realize automatic recognition of epilepsy signals (5). The methods for extracting signal features usually include three types: time-domain, frequency-domain, and time-frequency domain. For example, using wavelet transform, morphological analysis, and other methods to extract sample features. Then using methods such as Random Forest (RF) (6), Naive Bayes (NB) (7), Linear Discriminant Analysis (LDA) (8), and Support Vector Machine (SVM) (9) to classify the samples. Raghu et al. (10) used a combination of SVM and Continuous Decomposition Index for epilepsy detection. Omidvar et al. (11) used Discrete Wavelet Transform combined with Artificial Neural Networks with SVM for seizure detection. Ravi Kumar et al. (12) used variational mode decomposition combined with RF for automatic identification of EEG signals in epileptogenic regions. Sukriti et al. (13) proposed two multiscale entropy analysis methods, multiscale dispersion entropy (MDE) and refined composite multiscale dispersion entropy (RCMDE), which were used in combination with SVM for seizure detection with good results.

Deep learning involves feeding a large amount of data into a deep neural network for training and then using the trained neural network model to classify and predict the new data (14). Acharya et al. (15) first used Convolutional Neural Network (CNN) for seizure detection. The property of automatic feature extraction by Convolutional Neural Network significantly improved the performance of seizure detection compared to the traditional manual extraction of features (16). Aliyu et al. (17) proposed a Long Short Term Memory (LSTM) network for classifying epileptic EEG signals. Li et al. (18) proposed a patient-specific seizure prediction method based on Deep Residual Shrinkage Network (DRSN) and Gate Recurrent Unit (GRU). By introducing GRU into DRSN, they simulated the time dependence of signals in different time windows before the seizure. Hussain et al. (19) proposed an autonomous generalized retrospective and patient-specific hybrid model based on CNN and LSTM feature extractors.

Although existing deep learning-based methods for automatic epilepsy detection have made great progress, there are still two problems to be solved. One is that the correlation between EEG feature vectors is not taken into account and there is a lack of reconstructive enhancement of the features. The other is due to imbalanced data classification and insufficient sample size, resulting in poor recognition ability and unstable performance of the model. Based on the above two problems, this paper firstly adopts Adversarial and Mixup Data Augmentation (AMDA) method to train the model on the augmented training data DAMDA. Where DAMDA is obtained by adversarial training and mixup data augmentation. The augmented EEG data is then fed into the AM-1D CNN-GRU model for classification, which utilizes 1D CNN for high-dimensional feature extraction. The attention mechanism is introduced to enhance the correlation between the extracted features. Finally, GRU is utilized to fuse the information of the front and back sequences to fully integrate the information of the adjacent EEG signals and improve the accuracy of the model detection.

## 2 Materials and methods

### 2.1 Adversarial and mixup data augmentation

**Figure 1:**
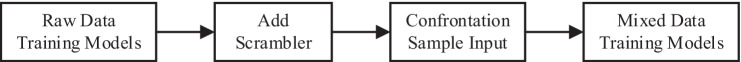
AMDA flowchart.

To better generalize the deep learning model and improve the robustness of the model to adversarial samples and noise from damaging EEG signals, this paper uses AMDA method to train the model adversarial. Christian Szegedy et al. (20) proposed the concept of admissible samples, that is, the input samples formed by deliberately adding subtle disturbances to the data set, and the disturbed inputs lead to the model giving an incorrect output with high confidence. By applying the Adversarial and Mixup Data Augmentation (AMDA) method, it effectively promotes the generalization ability of the deep learning model and significantly improves the robustness of the model in the face of adversarial samples as well as destructive noise. The AMDA method used in this paper is accomplished through the following steps, as shown in Figure 1.

Firstly, the network classifier is trained using raw EEG data, and then perturbations are added to the raw data. Input the generated adversarial samples into the classifier for classification, and obtain the classification error. Mixing the adversarial and original samples as the training set for the classifier to train. In the AMDA method used in this paper, the idea of generating perturbations is to compute the loss function $J\left(\theta ,x,y\right)$ of the deep neural network model, and the perturbations to be added by maximizing the loss function, where x and y are the original data and the corresponding real labels, and θ is a parameter of the model. The goal is to generate an adversarial sample $\bar{x}$ that is not easily distinguishable from x by maximizing $J\left(\theta ,x,y\right)$ to deceive the model, i.e., to misclassify using the adversarial sample $\bar{x}$ . Therefore, adversarial sample generation can be transformed into an optimization problem with the following constraints, as shown in Equation (1):


(1) $$\arg\;\max\;J\left(\theta \text{,,}\bar{x}\text{,,}y\right)s.t.∥\bar{x}-x∥{\;}_{\infty }<\varepsilon$$

### 2.2 Am-1D CNN-GRU network architecture

**Figure 2:**
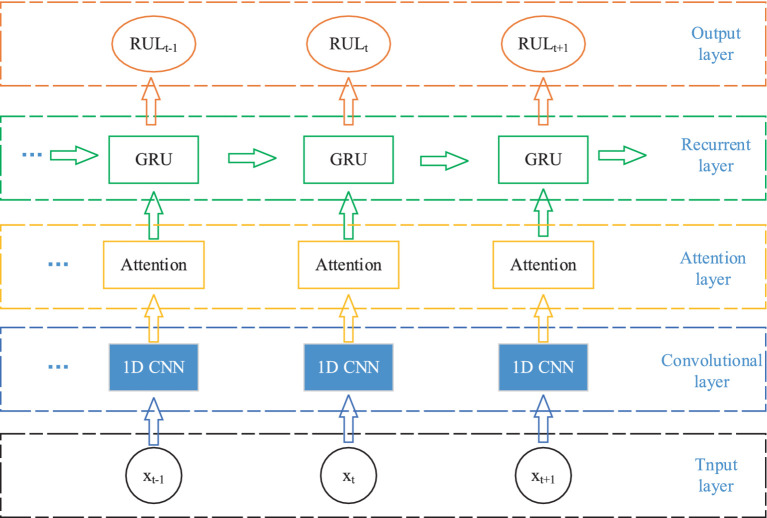
AM-1D CNN-GRU network structure.

The AM-1D CNN-GRU network proposed in this paper is divided into five parts, input layer, convolutional layer, attention layer, loop layer, and output layer. The specific structure is shown in Figure 2. The model does not require complex manual extraction of features, and compared with other models, the model designed in this paper has only 5 layers of network, with a small number of parameters, a simple model, and a small amount of computation, which can be well ported to mobile devices for practical applications in the later stage.

Firstly, the original EEG signal slices are input into the 1D CNN network for feature extraction, and the attention mechanism is utilized to assign weights to the feature vectors to highlight the important features. Then it is inputted into GRU network to fuse the information of the front and back sequences to fully integrate the information of the neighboring EEG signals. Finally, it is input to the full connectivity layer for classification.

#### 2.2.1 1D CNN models

**Figure 3:**
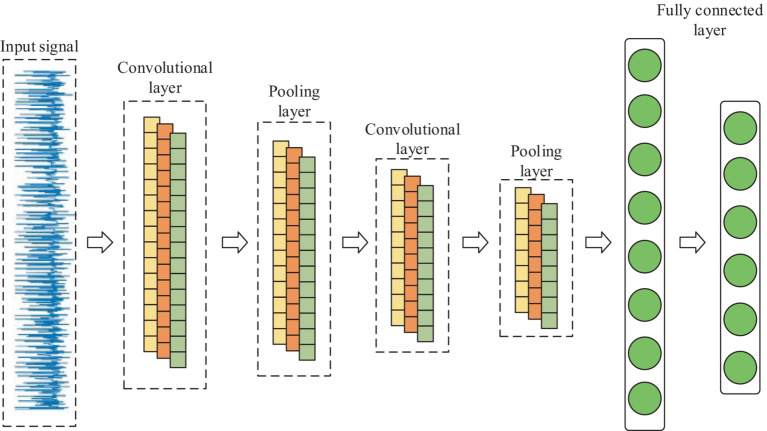
Internal structure of 1D CNN.

CNN is one of the representative algorithms for deep learning. Artificial neurons can respond to surrounding units and perform large-scale data processing, which maps the original inputs to new features through data transformation as well as dimensionality reduction (21). Unlike standard fully connected networks, CNN has a special network structure. It includes a feature extractor consisting of a convolutional layer and a pooling layer, which makes the network model simple by using local connectivity and weight sharing to extract features from the original data. Thus, it speeds up the training and improves the generalization performance (22). Because the EEG signal is a one-dimensional time series, 1D CNN is chosen in this paper. Its internal is shown in Figure 3.

In terms of network structure, 1D-CNN is the same as CNN, which contains 1D convolutional layer, 1D pooling layer, and fully connected layer, etc. Its main part usually consists of multiple 1D convolutional layers cascaded alternately with 1D pooling layer, which performs feature extraction on the input 1D data through multiple convolutional and pooling operations, and then classifies the input data through fully connected layer.

#### 2.2.2 Attention mechanism

**Figure 4:**
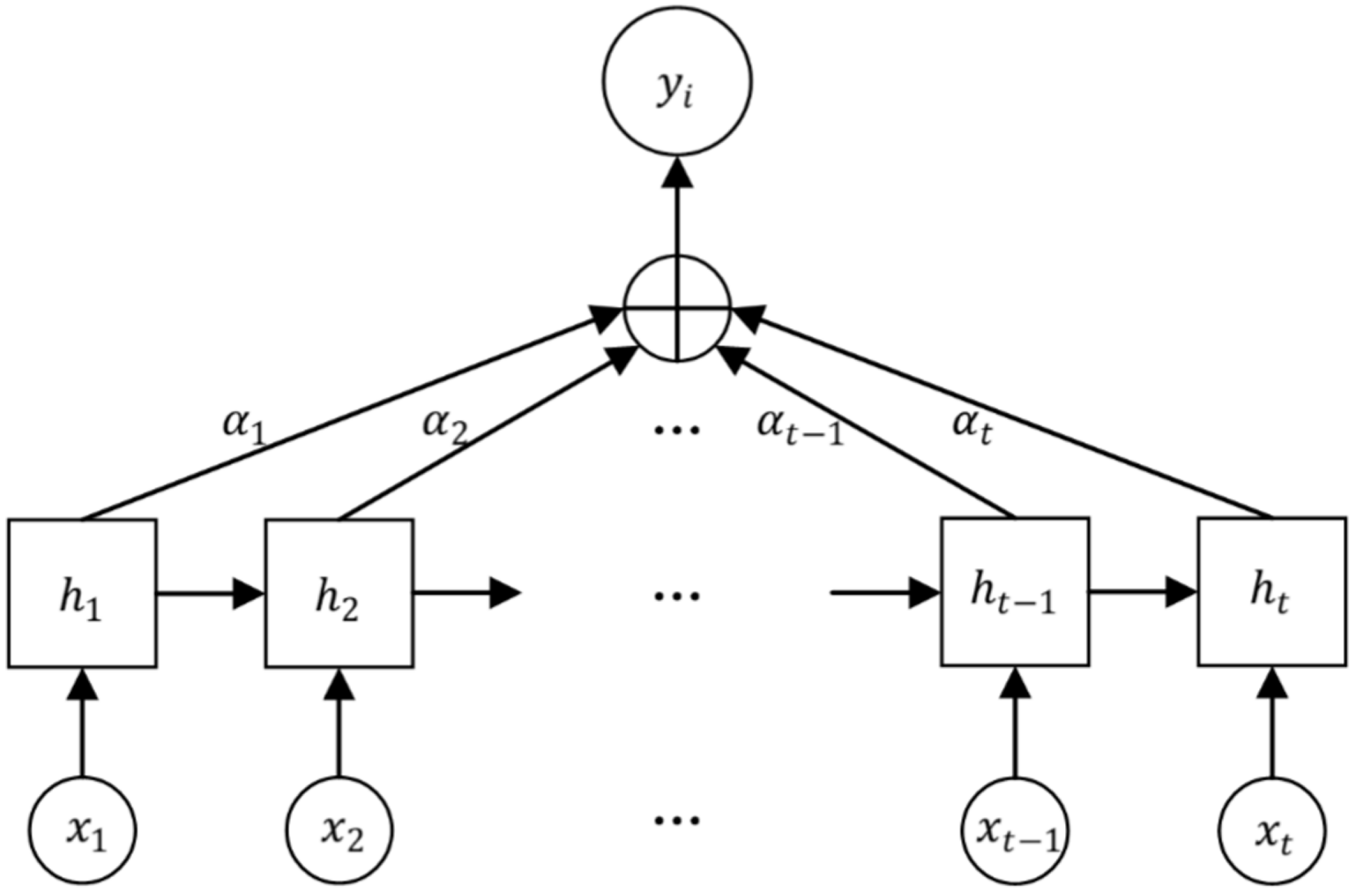
Attention structure.

The Attention mechanism is a mechanism that mimics the allocation of attention in the human brain. It is able to focus attention on important areas at a given moment and ignore or diminish attention to other areas. Thus, more detailed information is obtained and useless information is filtered out. Its core idea is to flexibly and reasonably adjust the attention to information, amplify the needed information, and suppress irrelevant information (23). Attention mechanism gives higher weight to key information through the method of probability allocation, highlighting the impact of important information, so as to improve the accuracy of the model. The structure of the Attention mechanism is shown in Figure 4.

**Figure 5:**
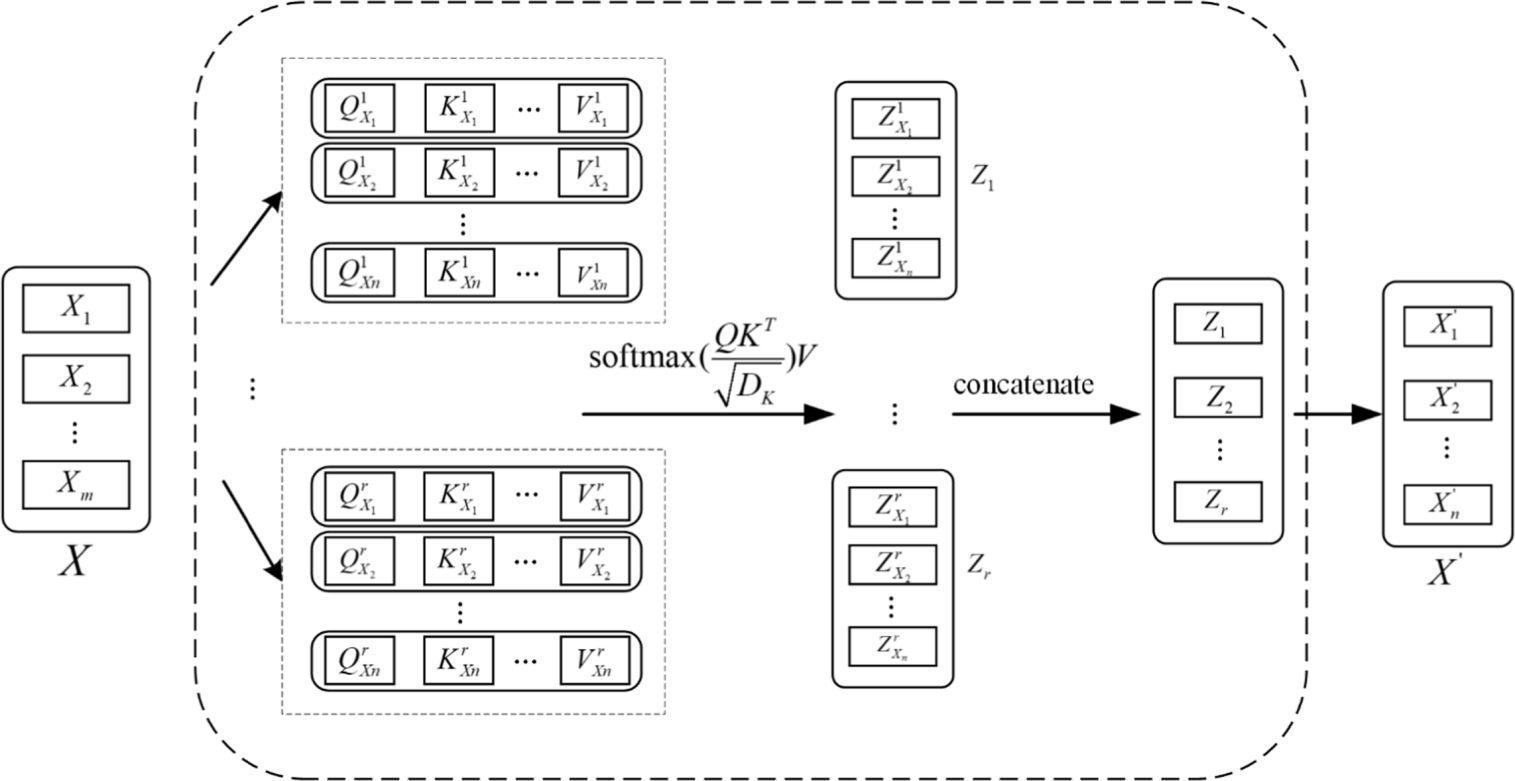
Feature reconstruction map.

The EEG signal is reconstructed for each feature vector by considering the connectivity between the individual feature vectors as it passes through the Attention layer, which processes the localized feature vectors of the input. The specific feature reconstruction process is shown in Figure 5.

Each local feature vector of the input creates three corresponding vectors, which are Query vector, Key vector and Value vector. These 3 vectors are extracted by the feature extraction module for each feature. These three vectors are each feature vector extracted by the feature extraction module and the three weight matrices $W^{Q}$ , $W^{K}$ , and $W^{V}$ .

#### 2.2.3 GRU model

**Figure 6:**
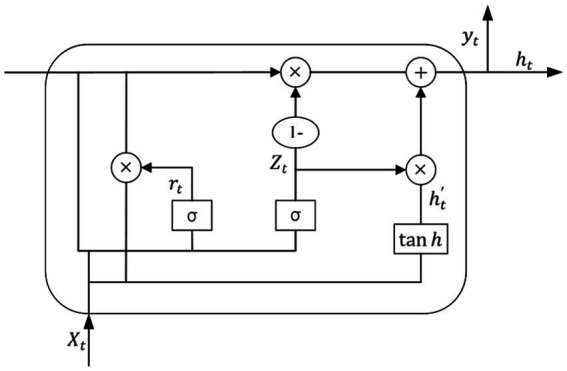
Basic unit of GRU network.

GRU is a variant of LSTM network, which has a simpler structure and can solve the problem of gradient explosion and disappearance of RNN in practical applications, and is widely used in the prediction of time series. GRU has a similar data flow with LSTM, but GRU lacks a separate storage unit, which makes it more efficient in the training process (24). There are only two gates in the GRU model: the update gate and the reset gate, and its specific structure is shown in Figure 6.

Where × is the number product of the matrix, σ is the Sigmoid function, tanh is the activation function, and 1− denotes that the data propagated forward by this link is $1-z_{t}$ . The update and reset gates are $z_{t}$ and $r_{t}$ respectively, $x_{t}$ is the input and $h_{t}$ is the output of the hidden layer.

## 3 Results

### 3.1 Dataset

Currently, two international open-source datasets widely used in epilepsy detection research are the University of Bonn epilepsy dataset and the Children’s Hospital Boston epilepsy dataset (CHB-MIT). However, the University of Bonn Epilepsy EEG dataset records single-channel EEG signals, which does not allow us to obtain important information about EEG signals between multiple channels. At the same time, the dataset has a very small amount of data and cannot well simulate the EEG recordings of real-life epileptic patients. Therefore, the CHB-MIT dataset is chosen to test the performance of the proposed model in this paper.

The CHB-MIT dataset consists of scalp EEG recordings of recalcitrant seizures in pediatric subjects with refractory epilepsy. All EEG data were collected using the international standard 10–20 EEG electrode position system, with a sampling frequency of 256 Hz. This dataset contains EEG records of 24 patients with epilepsy, with patient numbers 1 and 21 being the same patient and EEG data collection interval of one and a half years. Number 24 is supplementary data without detailed patient information. The entire database totaled up to 967.85 h of continuous scalp EEG recordings and 178 seizure events. Each case contained 18 or 23 multilead EEG recordings ranging from 9 to 42 (25). Due to the inconsistency of sampling channels for each subject, 13 patients with consistent channels were selected for experimental data in this paper. Details of the Boston Children’s Hospital scalp epilepsy EEG dataset are shown in Table 1.

**Table 1:** Dataset information.

Patient number	Gender	Age	Seizure/s	Non-seizure/s
1	Female	11	420	12,600
2	Male	11	164	118,800
3	Female	14	384	111,600
4	Male	22	368	532,800
5	Female	7	544	122,400
6	Female	1.5	120	147,600
7	Female	14.5	316	216,000
8	Male	3.5	908	54,000
9	Female	10	216	212,400
10	Male	3	380	129,600
11	Female	12	796	115,200
12	Female	2	1,372	36,000
13	Female	3	504	82,800
14	Female	9	148	61,200
15	Male	16	1846	93,600
16	Female	7	56	46,800
17	Female	12	288	61,200
18	Female	18	248	104,400
19	Female	19	232	97,200
20	Female	6	268	82,800
21	Female	13	192	97,200
22	Female	9	196	100,800
23	Female	6	400	68,400
24	-	-	484	36,000

### 3.2 Experimental process

**Figure 7:**
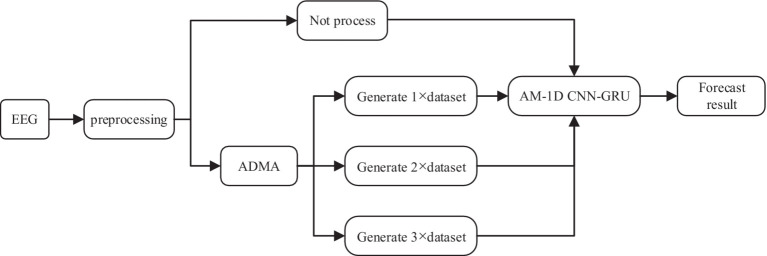
Experimental flow chart.

The experimental process in this paper mainly includes three parts: data preprocessing, data augmentation, and feature extraction and classification. The whole experimental flow is shown in Figure 7.

In the data preprocessing stage, most of the data sampling time is 1 h, too long data is not conducive to the network learning data feature information. Therefore, the scalp EEG signal is segmented into small equal length segments of data, and a segmentation time of 30 s was chosen for this experiment to ensure the smoothness of the sample. At the same time, the computation of the model can be largely reduced to ensure the real-time performance of the system. In the data augmentation stage, the preprocessed data were processed with no processing and AMDA algorithm respectively, in order to verify how much generated data can be added to the original dataset to optimize the performance of the classifiers, the adversarial samples of n times the original training set (n × dataset) were added to each patient using AMDA respectively, where n is 1, 2, and 3, and the data processed by the four methods were inputted into the AM-1D CNN-GRU model, respectively, to carry out the feature extraction and classification, and the classification performances of the four methods were compared. 70% of the dataset is used as a training set after the data augmentation process, and the other 30% is used as a test set to test the classification ability of the model.

### 3.3 Evaluation indicators

In this paper, we use accuracy, sensitivity specificity, and AUC as performance indicators, and each evaluation index is defined as follows.

1. Accuracy is defined as the proportion of correctly categorized samples to all samples and is calculated as shown in Equation (2):

(2) $$ACC=\frac{TP+TN}{TP+TN+FP+FN}$$

1. Sensitivity is defined as the proportion of correctly categorized positive samples to all positive samples, i.e., the proportion correctly judged to be ill, and is calculated as shown in Equation (3):

(3) $$SEN=\frac{TP}{TP+FN}$$

1. AUC is defined as the area under the subject’s work characteristic (ROC) curve, which indicates the probability that a pair of positive and negative samples are randomly selected and the model scores the positive samples greater than the negative samples. AUC ranges from 0.5 to 1 and is a performance indicator of the effectiveness of the classifier. The formula is as shown in Equations (4)–(6):

(4) $$TPR=\frac{TP}{TP+FN}$$

(5) $$TPR=\frac{TP}{TP+FN}$$

(6) $$AUC=\frac{1}{2}\overset{n-1}{\underset{i=1}{\sum }}\left(x_{i+1}-x_{i}\right)\left(y_{i}+y_{i+1}\right)$$

Where TP is a positive case that was correctly diagnosed as a positive case, TN is a negative case that was correctly diagnosed as a negative case, FP is a negative case that was incorrectly diagnosed as a positive case, FN is a positive case that was incorrectly diagnosed as a negative case. FPR (False Positive Rate) is the horizontal coordinate of the ROC curve, TPR (True Positive Rate) is the vertical coordinate, and, denote the successive coordinates on the ROC curve, respectively.

### 3.4 Experimental parameters

The hyperparameter settings for model training have a great impact on the prediction effect of the model. In order to make the CNN fully extract the features of the dataset, the size of the convolutional kernel of the 1D convolutional layer is set to 3 × 1, maximum pooling is chosen, and the number of GRU neurons is set to 128. Since the GRU cannot directly output the RUL, the fully-connected layer has to be added to predict the RUL, and a Dropout layer is added to the network to prevent overfitting, and the size of Dropout is set to 0.5. Finally, the learning rate is chosen to be 0.001, and the optimizer is chosen to be Adam and padding = same, and the training rounds are 50 times. Finally, the learning rate size is chosen as 0.001, the optimizer is chosen as Adam and padding = same is chosen for padding, the number of training rounds is 50 and the Batch Size is set to 256.

### 3.5 Experimental results

Four different methods were used to process the training set and the classifier was tested on each case. The results are shown in Table 2.

**Table 2:** Classification results of different data enhancement methods.

Method	Performance indicators		
	Accuracy	Sensitivity	AUC
Not process	84.73%	82.89%	0.8813
1 × dataset	96.06%	95.48%	0.9637
2 × dataset	93.28%	92.36%	0.9422
3 × dataset	89.16%	88.94%	0.9039

As can be seen from Table 2, compared with the classification results of unprocessed data, AMDA algorithm has significantly improved the classification results. The reason is that the nature and characteristics of AMDA are used to apply adversarial samples to data augmentation, which improves the amount of training data. The reason is that the use of the properties and characteristics of AMDA and the application of confrontation samples in data augmentation have improved the amount of training data. The AMDA algorithm is used to amplify the original data set by 1, 2, and 3 times, and the results show that when the amplified sample is 1 × dataset, the classification effect is slightly better than 2 × dataset and 3 × dataset. When more adversarial samples were continually added, the model’s classification effectiveness decreased, suggesting that as the number of adversarial samples increased, so did the number of features that were irrelevant to epilepsy detection, and the classification effectiveness was limited as a result.

## 4 Discussion

**Figure 8:**
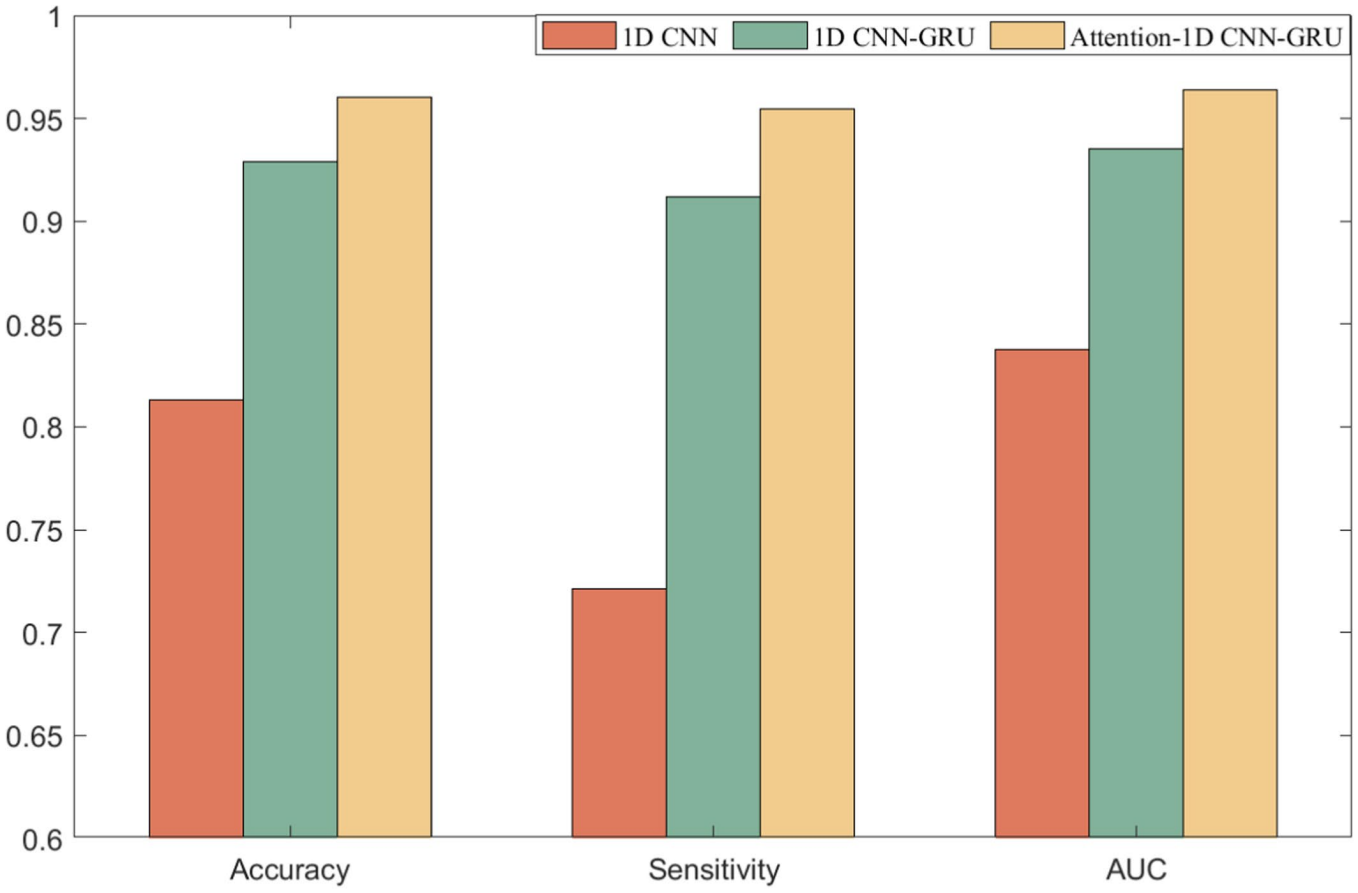
Comparison of experimental results.

The model proposed in this paper consists of three parts: 1D CNN, Attention mechanism, and GRU. Although the above three parts have great advantages in their respective fields, however, in the seizure detection work, only one or two of them cannot fully highlight their advantages, and only a clever combination of the above three parts can accomplish the seizure detection work. It can be seen from the results that the performance of the AM-1D CNN-GRU model proposed in this paper is better than that of the model without attention mechanism, which proves that this model has a relatively excellent seizure detection ability. The comparative experimental results are shown in Figure 8.

In the selection of comparison literature, the literature (26–36) take CHB-MIT dataset as the test and validation object. Therefore, the method of this paper will be compared with these literatures to verify the superiority of the method of this paper, and the experimental comparison results are shown in Table 3. In the comparison experiment, the method proposed in this paper is analyzed with the experimental results when the amplified sample is 1 × dataset in comparison with the comparison literature.

**Table 3:** Classification performance of different methods on the CHB-MIT EEG dataset.

Method	Number of patients	Accuracy	Sensitivity	AUC
RF (26)	23	86.73%	76.08%	-
SVM (27)	-	-	94.21%	-
GMM (28)	6	86.93%	86.26%	-
IndRNN (29)	18	88.70%	88.80%	-
LSTM (30)	-	83.89%	83.72%	-
DWT + SVM (31)	18	89.03%	91.71%	-
CNN + MIDS (32)	23	-	74.08%	-
MPE-ANN (33)	13	87.64%	87.27%	-
Attention+CNN (34)	16	94.42%	93.09%	-
Bi-LSTM (35)	24	93.61%	91.85%	-
LRCN (36)	23	99.00%	84.00%	-
Proposed method	13	96.06%	95.48%	0.9637

Previous studies have shown that deep learning has better performance than traditional methods for automatic seizure detection. In this chapter, RF and SVM, which are the better performing traditional machine learning classifiers, were chosen for comparison. The experimental results in Table 3 also verify this conclusion. At the same time, this chapter also compares the proposed method with some deep learning based classifiers. Literature (32) proposes an automatic epilepsy detection classification model based on convolution and attention mechanisms, and the experimental results of this model are the closest to the method proposed in this paper. An 18-layer long-term recursive convolutional network model was constructed in the literature (34) and achieved results with a sensitivity of 84% and an accuracy of 99%, which is higher than the final results of this paper, but the sensitivity is lower than the 95.48% of this paper, which is usually a more critical evaluation metric for epileptic seizure detection. Analyzing the superiority of the proposed method in this paper, there are three aspects: (1) AMDA is used for data augmentation, which increases the availability and effectiveness of the dataset. (2) Give higher weight to key information through Attention mechanism to highlight the impact of important information. (3) Using GRU to fuse the information of the front and back sequences fully integrates the information of the neighboring EEG signals and improves the performance of the model detection.

In order to test the robustness of the model against noisy or artifact-laden EEG data, epilepsy detection was implemented in non-denoised signals. The model proposed in this paper achieves an experimental result of accuracy 87.65%, sensitivity 88.72%, and AUC 0.8859. It can be seen that the performance obtained for the denoising case is higher than that obtained for the non-denoising case. Although the non-denoised EEG signals will retain more information, the noise contained in them will affect the judgment of the classifier, which will result in misjudgment and affect the performance of the epilepsy detection model.

The model proposed in this paper can be transplanted to the epilepsy detection clinical diagnosis and treatment equipment to help doctors understand more about the patient’s physical condition, so as to formulate the treatment plan in a targeted way, avoiding some unnecessary physical examinations that may bring harm to the body, which the doctor makes the patient do in order to determine the cause of the disease. At the same time, some of the early symptoms of epilepsy patients are relatively hidden, resulting in many patients ignoring them. The model proposed in this paper can assist doctors in making accurate diagnosis of epilepsy, so that patients can take preventive measures and reduce the pain caused by the onset of epilepsy.

## 5 Conclusion

In this paper, epilepsy detection is realized using EEG signals and data augmentation of epileptic EEG data using AMDA under the condition of limited number of training samples. The AM-1D CNN-GRU model is used for classification and the performance of the method is tested on the CHB-MIT dataset. Combining the two methods yielded more ideal results, with accuracy, sensitivity, and area under the subject’s work characteristic curve reaching 96.06, 95.48%, and 0.9637, respectively, when the amplified sample was 1 × dataset. Comparison with related literature shows superiority over many existing classification methods. The method proposed in this paper is not only important for detecting epileptic seizures, but also provides a very effective method for classifying time series in other fields. At this stage, this experiment is tested on a public dataset, and in future work, the validity of the proposed method will be further validated on more clinical EEG datasets to improve the model detection performance and to explore the available high-precision, high-adaptive, and high-reliable algorithms for the clinical application of epilepsy detection.

## Data availability statement

Publicly available datasets were analyzed in this study. This data can be found at: PhysioNet, https://physionet.org/, DOI: 10.13026/C2K01R.

## Ethics statement

Ethical review and approval was not required for the study on human participants in accordance with the local legislation and institutional requirements. Written informed consent from the patients/participants or patients/participants legal guardian/next of kin was not required to participate in this study in accordance with the national legislation and the institutional requirements.

## Author contributions

YR: Conceptualization, Formal analysis, Funding acquisition, Software, Validation, Writing – review & editing. ZW: Conceptualization, Methodology, Writing – original draft. GA: Conceptualization, Validation, Writing – review & editing. HC: Project administration, Supervision, Writing – review & editing.

## References

[1] Mahmoodian MHJ. Epileptic seizure detection using cross-bispectrum of electroencephalogram signal. Seizure. (2019) 66:4–11. doi: 10.1016/j.seizure.2019.02.001, PMID: 30769009

[2] Stevens JR. Seizure occurrence and Interspike interval. JAMA Neurol. (1972) 26:409–19. doi: 10.1001/archneur.1972.00490110043004 4337303

[3] Aksa B Gbbb C Ma A. An intelligent learning approach for improving ECG signal classification and arrhythmia analysis. Artif Intell Med. (2022) 103:101788. doi: 10.1016/j.artmed.2019.101788 32143795

[4] Sharma R Pachori RB. Classification of epileptic seizures in EEG signals based on phase space representation of intrinsic mode functions. Expert Syst Appl. (2015) 42:1106–17. doi: 10.1016/j.eswa.2014.08.030

[5] Yongfeng QI Xiaoxu PEI Xuechao LV. Epileptic EEG signal detection method based on synchronous compression and DCGAN. Comput Eng Sci. (2022) 44:2273–80.

[6] Birjandtalab J Pouyan MB Cogan D. Automated seizure detection using limited-channel EEG and non-linear dimension reduction. Comput Biol Med. (2017) 82:49–58. doi: 10.1016/j.compbiomed.2017.01.011, PMID: 28161592

[7] Diykh M Li Y Wen P. Classify epileptic EEG signals using weighted complex networks based community structure detection. Expert Syst Appl. (2017) 90:87–100. doi: 10.1016/j.eswa.2017.08.012

[8] Yuan S Liu J Shang J. The earth mover's distance and Bayesian linear discriminant analysis for epileptic seizure detection in scalp EEG. Biomed Eng Lett. (2018) 8:373–82. doi: 10.1007/s13534-018-0082-3 30603222 PMC6209084

[9] Richhariya B Tanveer M. EEG signal classification using universum support vector machine. Expert Syst Appl. (2018) 106:169–82. doi: 10.1016/j.eswa.2018.03.053

[10] Raghu S Sriraam N Rao SV. Automated detection of epileptic seizures using successive decomposition index and support vector machine classifier in long-term EEG. Neural Comput Applic. (2019) 32:8965–84. doi: 10.1007/s00521-019-04389-1

[11] Omidvar M Zahedi A Bakhshi H. EEG signal processing for epilepsy seizure detection using 5-level Db4 discrete wavelet transform, GA-based feature selection and ANN/SVM classifiers. J Ambient Intell Humaniz Comput. (2021) 4:1–9. doi: 10.1007/s12652-020-02837-8

[12] Ravi KM Srinivasa RY. Epileptic seizures classification in EEG signal based on semantic features and variational mode decomposition. Clust Comput. (2019) 22:13521–31. doi: 10.1007/s10586-018-1995-4

[13] Sukriti Chakraborty M Mitra D. Automated detection of epileptic seizures using multiscale and refined composite multiscale dispersion entropy. Chaos Solitons Fractals. (2021) 110939:0960–779. doi: 10.1016/j.chaos.2021.110939

[14] Zhang H Ma J Zhang N. Advances in deep learning for epilepsy detection. Comput Eng Appl. (2023) 59:35–47.

[15] Acharya UR Oh SL Hagiwara Y Tan JH Adeli H. Deep convolutional neural network for the automated detection and diagnosis of seizure using EEG signals. Comput Biol Med. (2018) 100:270–8. doi: 10.1016/j.compbiomed.2017.09.017, PMID: 28974302

[16] Yu Z Nie W Zhou W. Epileptic seizure prediction based on local mean decomposition and deep convolutional neural network. J Supercomput. (2020) 76:1–15. doi: 10.1007/s11227-018-2600-6

[17] Aliyu I Lim CG. Selection of optimal wavelet features for epileptic EEG signal classification with LSTM. Neural Comput & Applic. (2021) 35:326–49. doi: 10.1007/s00521-020-05666-0

[18] Li L Zhang H Liu X. Detection method of absence seizures based on Resnet and bidirectional GRU. Acta Epileptologica. (2023) 5:132–41. doi: 10.1186/s42494-022-00117-w PMC11960378 40217463

[19] Hussain W Sadiq MT Siuly S Rehman AU. Epileptic seizure detection using 1 D-convolutional long short-term memory neural networks. Appl Acoust. (2021) 177:107941:107941. doi: 10.1016/j.apacoust.2021.107941

[20] Szegedy C Zaremba W Sutskever I. Intriguing properties of neural networks. arXiv. (2013). doi: 10.48550/arXiv.1312.6199

[21] Lee SY Hung YW Chang YT. RISC-V CNN coprocessor for real-time epilepsy detection in wearable application. IEEE Trans Biomed Circuits Syst. (2021) 15:679–91. doi: 10.1109/TBCAS.2021.3092744 34181550

[22] Zhang R Feng G Fu H. Research on blueberry shelf-life prediction model based on CNN-GRU-AE. Food Science. (2023) 1:15.

[23] Lebal A Moussaoui A Rezgui A. Epilepsy-net: attention-based 1D-inception network model for epilepsy detection using one-channel and multi-channel EEG signals. Multimed Tools Appl. (2022) 82:17391–413.

[24] Fang Z Leung H Choy CS. Spatial temporal GRU convnets for vision-based real time epileptic seizure detection. Abbreviated J Name. (2018) 4:1026–9. doi: 10.1109/ISBI.2018.8363746

[25] Jun J. Research on EEG signal analysis algorithm for epilepsy warning task. Changchun: Jilin University (2021).

[26] Xuelan H. Research on seizure detection method based on abnormal wave of scalp EEG signal. Hangzhou: Zhejiang University of Technology (2023).

[27] Raghu S Sriraam N Temel Y Rao SV Hegde AS Kubben PL. Performance evaluation of DWT based sigmoid entropy in time and frequency domains for automated detection of epileptic seizures using SVM classifier. Comput Biol Med. (2019) 110:127–43. doi: 10.1016/j.compbiomed.2019.05.016, PMID: 31154257

[28] Zuo R Wei J Li X. Automated detection of high-frequency oscillations in epilepsy based on a convolutional neural network. Front Comput Neurosci. (2019) 13:6–15. doi: 10.3389/fncom.2019.00006, PMID: 30809142 PMC6379273

[29] Yao X Cheng Q Zhang GQ. Automated classification of seizures against nonseizures: a deep learning approach. IEEE Trans Biomed Eng. (2019) 9:31–40. doi: 10.48550/arXiv.1906.02745

[30] Yao X Li X Ye Q. A robust deep learning approach for automatic classification of seizures against non-seizures. Biomed Signal Proces Control. (2021) 64:102215. doi: 10.1016/j.bspc.2020.102215

[31] Duo C Suiren W Jing X. A high-performance seizure detection algorithm based on discrete wavelet transform (DWT) and EEG. PLoS One. (2017) 12:0173138. doi: 10.1371/journal.pone.0173138 PMC5344346 28278203

[32] Wei Z Zou J Zhang J. Automatic epileptic EEG detection using convolutional neural network with improvements in time-domain. Biomed Signal Proces Control. (2019) 53:101551. doi: 10.1016/j.bspc.2019.04.028

[33] Yufei S Yao W. Classification study of epilepsy EEG detection based on MPE-ANN-SVM. J Biomed Eng Res. (2022) 4:41. doi: 10.19529/j.cnki.1672-6278.2022.04.01

[34] Xin D Hongwei G. Epilepsy EEG detection model based on convolution and attention mechanism. J Chongqing Univ Posts Telecommun. (2023) 35:927–34.

[35] Hu X Yuan S Xu F. Scalp EEG classification using deep bi-LSTM network for seizure detection. Comput Biol Med. (2020) 124:103919. doi: 10.1016/j.compbiomed.2020.103919, PMID: 32771673

[36] Liang W Pei H Cai Q. Scalp EEG epileptogenic zone recognition and localization based on long-term recurrent convolutional network. Neurocomputing. (2020) 396:596–76. doi: 10.1016/j.neucom.2018.10.108

